# Review of the Effects and Mechanism of Curcumin in the Treatment of Inflammatory Bowel Disease

**DOI:** 10.3389/fphar.2022.908077

**Published:** 2022-06-20

**Authors:** Yuan Lin, Hengjian Liu, Lingling Bu, Chen Chen, Xiaofeng Ye

**Affiliations:** Changzhou Hospital of Traditional Chinese Medicine, Changzhou, China

**Keywords:** IBD, curcumin, dietary supplements, immune regulation, anti-inflammatory actvity

## Abstract

Curcumin is extracted from the rhizomes of *Curcuma longa L*. It is now widely used in food processing, cosmetics, dyes, etc. Current researching indicates that curcumin has high medical value, including anti-inflammatory, antioxidant, anti-tumor, anti-apoptotic, anti-fibrosis, immune regulation and other effects, and can be used to treat a variety of diseases. Inflammatory bowel disease (IBD) is a nonspecific inflammatory disease of the intestine including Crohn’s disease (CD) and ulcerative colitis (UC). The drug treatment effect is often limited and accompanied by side effects. A large number of basic and clinical studies have shown that curcumin has the effect of treating IBD and also can maintain the remission of IBD. In this review, the research of curcumin on IBD in recent years is summarized in order to provide reference for further research and application of curcumin.

## 1 Introduction

**FIGURE 1:**
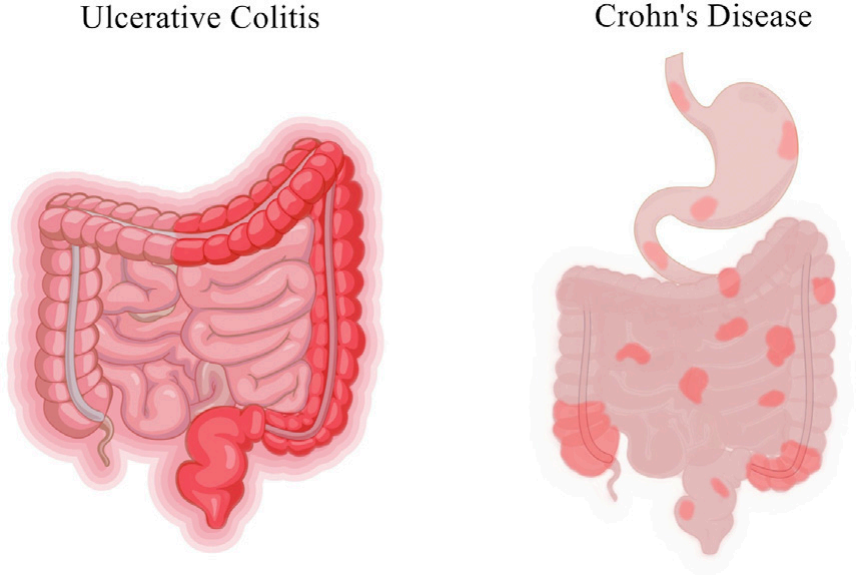
The common affected parts of IBD.

Inflammatory bowel disease (IBD) is a nonspecific inflammatory disease of the intestine with unknown etiology, including Crohn’s disease (CD) and ulcerative colitis (UC). The lesions of CD mainly involve the terminal ileum and proximal colon, and the lesions of UC are mostly accumulated in the rectum and part of the colon (Baumgart and Sandborn, 2012; Ungaro et al., 2017) (Figure 1). In recent years, the prevalence of IBD showed an upward trend year by year. In the United States alone, there are about 70,000 new confirmed cases each year (Colombel and Mahadevan, 2017). Although the prevalence in Asia and Africa is lower than that in western developed countries, it also shows an overall upward trend. Moreover, the prevalence and incidence of IBD in children are gradually increasing worldwide. According to statistics from the Chinese Center for Disease Control and Prevention, the number of IBD patients in China might increase to 1.5 million by 2025. IBD is a chronic recurrent disease, and patients often require lifelong medication to control clinical symptoms, alleviate pathological damage such as mucosal barrier destruction, and reduce recurrence. IBD is often accompanied by intestinal pathological changes, characterized by intestinal inflammation and damage to the intestinal mucosal barrier (van der Veen et al., 2009). The most common symptoms of IBD include abdominal pain, diarrhea, bowel obstruction and weight loss (Table 1). IBD is considered to be the result of the continuous inflammatory process against endogenous microbes in genetically susceptible individuals. The existing studies indicated that IBD outcome is a result of a complex interplay between genetic, immunologic, and modifiable environmental factors in a genetically susceptible host against the subset of gut commensal microbiota (Castro et al., 2015). The rising incidence of IBD indicates the necessity of studying the environmental factors. In addition, clearing environmental factors can help prevent and treat the disease.

**TABLE 1:** The proportion of the clinical symptoms of IBD (Li et al., 2017; Annese, 2019; Rogler et al., 2021).

	Symptoms	Crohn’s disease	Ulcerative colitis
Gastrointestinal Symptoms	Abdominal pain	63–79.4%	37.7–64.9%
	Diarrhoea	36.4–54.2%	72.8–83.7%
	Mucopurulence and bloody stool	16.3%	74.2%
	Anal fistula and perianal abscess	19.6%	—
General Manifestations	Anaemia	24.8%	13.4%
	Fever	32%	18.2%
	Weight loss	44.4%	28.4%
Extra-intestinal Manifestations	Peripheral arthritis	10–20%	4–14%
	Metabolic bone disease	20–50%	—
	Primary sclerosing cholangitis	<1%	4–5%
	Anterior uveitis	5–12%	3.5–4.1%
	Erythema nodosum	5–15%	2–10%
	Oral aphthous ulcers	5–50%	<1%

The effect of medicinal treatment is often affected by the compliance and economic status of patients (Khan et al., 2012). At present, the drug effect is limited, the price is high, and often accompanied by side effects (i.e., diarrhea and lymphopenia). IBD is difficult to delay to heal and easy relapse. The economic burden of patients is heavy, and the quality of life is often seriously affected. Researchers have been trying to find plant-derived products or dietary supplements that could treat IBD.

Curcumin is an orange-yellow polyphenolic chemical substance extracted from the rhizomes of *Curcuma longa L*. It is the main active ingredient of Chinese medicine Curcuma Longa and is now widely used in food processing, cosmetics, dyes, etc. Curcumin has been used as a traditional herbal medicine in India and Southeast Asia for thousands of years. It is often used to treat biliary tract diseases, anorexia, rhinitis, cough, rheumatism, and various chronic inflammatory diseases. Due to its extensive biological activities, it has received widespread attention from researchers in recent years. Current researching indicates that curcumin has high medical value, including anti-inflammatory, antioxidant, anti-tumor, anti-apoptotic, anti-fibrosis, immune regulation and other effects, and can be used to treat a variety of diseases (Salehi et al., 2019). It is involved in many significant genetic and biochemical pathways (Karthikeyan et al., 2020; Moniruzzaman and Min, 2020; Beyene et al., 2021). Curcumin is associated with many cellular targets (i.e., NF-κB, JAKs/STATs, MAPKs, TNF-*γ*, IL-6, PPAR*γ*, and TRPV1) that effectively reduce the progression of IBD. The research of curcumin and related formulations for IBD treatment has surged over the decade (Kahkhaie et al., 2019; Sharma et al., 2019; Patel et al., 2020). So far, a large number of basic and clinical studies have shown that curcumin has the effect of treating IBD and also can maintain the remission of IBD (Yang H. et al., 2017). In this review, the researches of IBD genetics and pathogenesis and curcumin molecular targets in IBD in recent years are summarized in order to provide reference for further research and application of curcumin.

## 2 Curcumin

### 2.1 Ingredient and Structure

**FIGURE 2:**
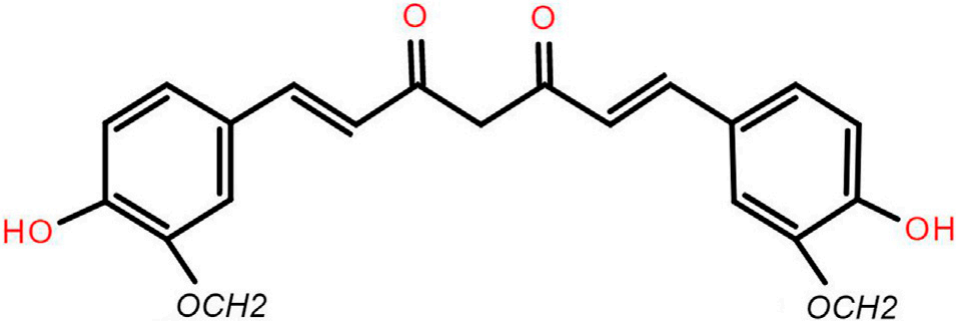
Chemical structure of curcumin.

The main components of curcumin are curcumin, demethoxycurcumin and bisdemethoxycurcumin, which are the main components of *Curcuma longa* to play the pharmacologic effects. Curcuminoid account for about 70% of the total content of curcumin. The backbone of the molecular formula of curcumin is unsaturated fatty hydrocarbons and aromatic group. It is easily soluble in methanol, ethanol, acetone, ethyl acetate and slightly soluble in benzene and ether. Curcumin has low solubility in water and is a strong photosensitive substance. The molecular formula of curcumin is C_21_H_20_O_6_ (Epstein et al., 2010a) (Figure 2).

### 2.2 Concentration and Absorption

Curcumin is almost insoluble in aqueous solution because of its lipophilic properties. It is not easy to be absorbed after oral administration. After absorption, curcumin is rapidly discharged from the body through metabolism. Its bioavailability in the body is low, thus affecting its use as a drug directly. Current research results showed that dosage form modification or drug combination can effectively increase the water solubility of curcumin and improve its bioavailability.

Ravindranath et al. administered 400 mg of curcumin orally to rats. After 24 h, the curcumin concentration in the lower part of the intestine (cecum, large intestine) was 38% of the administered dosage. Within to 24 h after oral administration of curcumin, the drug residues in liver and kidney tissue were less than 20 μg per gram of tissue (Ravindranath and Chandrasekhara, 1980). Pan et al. found that 0.1 g/kg body weight of curcumin was injected intraperitoneally into mice, the plasma curcumin content was about 2.25 mg/L after 15 min. The curcumin content in the intestine, spleen, liver and kidney was 177.04 μg/g, 26.06 μg/g, 26.90 μg/g and 7.51 μg/g respectively at 1 h. Only trace amounts (0.41 μg/g) were present in the brain (Pan et al., 1999). The results confirmed that the distribution of curcumin in the gastrointestinal tract and kidney is much larger than that in the liver and plasma. It may be because after oral administration, curcumin is mainly absorbed through the gastrointestinal tract, and is widely distributed in the liver and other organs after entering the blood.

### 2.3 Metabolism and Toxicity

Sharma et al. studied the pharmacokinetics of curcumin through oral administration of it in 15 patients with advanced colon cancer. Patients received curcumin at 180 mg orally and their plasma and urine were collected. HPLC method was used to determine the content of curcumin. While curcumin and its metabolites were almost not detected, but it was found in the feces of patients. The results showed that curcumin was safe and well tolerated at 180 mg per day orally (Sharma et al., 2001). Lao et al. used dose-escalation to study the maximum tolerated dose and safety of curcumin. Twenty-four healthy subjects took curcumin extract powder orally at doses ranging from 500 mg to 12,000 mg, and only seven of them had minor non-dose-related toxicity. Therefore, curcumin is well tolerated at a single oral dose up to 8000 mg (Lao et al., 2006). Cheng et al. conducted a prospective clinical study on 25 patients with five different malignant diseases to evaluate the safety of curcumin. The initial dose was 500 mg/d, and if there were no consecutive grade 2 or more toxicity, the dose was increased to 1000, 2000, 4000, and 8000 mg/d. The results indicated that there was no curcumin-related toxicity after oral administration of 8000 mg/d for 3 months (Cheng et al., 2001).

### 2.4 Therapeutic Effects

In recent years, studies have shown that curcumin has great pharmacological effects on anti-inflammatory, anti-fibrosis, anti-oxidation, hypolipidemic, anti-atherosclerosis, anti-depression, anti-HIV virus, etc. At the same time, curcumin has very low toxicity, so it has great potential for clinical application. Curcumin can also inhibit the growth of various tumor cells and prevent the occurrence of gastric cancer, duodenal cancer, colon cancer, breast cancer, chemical and radiation-induced skin cancer in rats. Its anti-tumor mechanism mainly includes inducing tumor cell apoptosis, blocking tumor cell signal transduction pathway, anti-oxidation and inhibiting tumor angiogenesis. For inflammatory bowel disease, curcumin can exert immunomodulatory and anti-inflammatory effects by inhibiting inflammatory mediators and cytokines, scavenging oxygen free radicals and other processes. This article will discuss the different mechanisms of curcumin in the treatment of inflammatory bowel disease.

## 3 Curcumin and Inflammatory Bowel Disease

### 3.1 *In vitro* and *in vivo* Experiment

#### 3.1.1 Effects on Inflammatory Factors

IBD is an autoimmune disease involving both autoantibodies and autoreactive CD4-positive T-lymphocytes. Dysimmunity will cause CD4-positive T-lymphocytes (Th1, Th2, and Th17) to produce a large number of pro-inflammatory factors, pushing the immune response beyond the range of T regulatory cells (Tregs), then triggering IBD. Jobin et al. demonstrated that curcumin inhibits il-*β*-mediated expression of pro-inflammatory cytokines such as IC-1 and IL-8 in IEC-6, HT-29 and Caco-2 cells, thereby exerting its anti-inflammatory effects (Jobin et al., 1999).

IL-1 mainly activates T cells, macrophages, etc. and its expression level increases significantly in the active period of IBD. Curcumin can inhibit the expression and secretion of inflammatory protein (MIP-2), IL-1β, and cytokine (KC) by macrophages stimulated by lipopolysaccharide (LPS). It blocks the accumulation of neutrophils to the site of intestinal inflammation, thereby alleviating the inflammatory response of IBD (Larmonier et al., 2011). IL-10 is an immunomodulatory cytokine synthesized by Th2, which can fight against pro-inflammatory factors such as IL-2, TNF-*α* and IFN-*γ*, and balance the body’s inflammatory response. The researchers found that the mouse model of colitis could be established using IL-10 gene knockout mice, demonstrating that IL-10 expression deficiency is associated with the pathogenesis of IBD (McFadden et al., 2015). Epstein et al. extracted colon tissue cells from IBD patients and used curcumin for intervention. The results showed that curcumin can inhibit the activation of p38MAPK, reduce the expression of IL-1β and matrix metalloproteinase-3 (MMP-3) and increase the expression of IL-10 (Epstein et al., 2010b). Curcumin inhibits LPS-induced expression of IL-12/23p40 in dendritic cells by synergistic action with IL-10 (Larmonier et al., 2008). Curcumin can regulate the expression of ALDH1a and IL-10 in bone marrow-derived dendritic cells (DCs). Curcumin-treated DCs can induce the differentiation of CD4^+^ T cells into Treg, which in turn inhibits the activation of antigen specific T cells. Thus, it promotes the restoration of immune balance. The results confirmed that curcumin has an effective therapeutic effect on experimental IBD (Cong et al., 2009).

IFN-*γ* is mainly produced by Th1 cells. Researchers found that IFN-*γ* levels in plasma and intestinal mucosa were significantly higher in IBD patients in active stage than in remission stage (Onal et al., 2016). Kiela et al. found that curcumin inhibited IFN-*γ* signaling in human and mouse colon epithelial cells. It plays a dual regulatory mechanism in colon epithelial cells, thereby improving and alleviating IBD (Midura-Kiela et al., 2012).

TNF-*α* is a major cytokine involved in inflammatory cascades in IBD. It has independent apoptotic activity and plays a key role in disruption of the intestinal barrier (Vedantam and Viswanathan, 2011). Curcumin also inhibits or kills B-lymphocytes, thereby blocking the release of macrophage-mediated inflammatory cytokines TNF-*α* and IL-6 (Maradana et al., 2013). Curcumin can down-regulate the production of Indoleamine 2,3-dioxygenase (IDO) through coX-2/PGE2 pathway. It inhibits the expression of surface molecules (CD80, CD86 and MHC class I) and proinflammatory cytokines (IL-12 P70 and TNF-*α*), thereby playing an immunomodulatory role (Jung et al., 2010). Yeter et al. found that curcumin can effectively inhibited TNF-*α* release and prevent TNF-*α* -driven oxidative stress response, thereby significantly reducing inflammatory response in mice with colitis (Mouzaoui et al., 2012).

#### 3.1.2 Effects on Transcription Factors

Regulation of Toll-like receptor 4 (TLR-4) is an intracellular pattern recognition receptor, which plays an important role in response to intestinal epithelial injury and in limiting intestinal bacterial migration in mice with colitis. Nuclear factor-κB (NF-κB) also plays an important role in the pathogenesis of IBD. A high level of NF-κB expression increased the capability to secrete cytokines (IL-1, IL-6, TNF-*α*, et al). It was associated with mucosal damage in IBD (Atreya et al., 2008). Inhibition of NF-κB activity has been researched as one of the main treatment methods for IBD. Through the TLR-4/MyD88/MAPK/NF-κB pathway, NF-κB can be activated and transferred to the nucleus, thereby inducing the expression of multiple inflammatory mediators, resulting in intestinal inflammatory injury. Curcumin inhibits NF-κB activation by inhibiting TLR-4 receptor activation and reduces cytoplasmic IκB protein degradation. At the same time, the expression level of MMP-9 was significantly reduced, and then the inflammatory cascade was stopped, the intestinal mucosal damage was relieved (Lubbad et al., 2009).

Peroxisomeproliferator-activated receptor *γ* (PPAR-*γ*), the member of the nuclear receptor superfamily, is expressed at high level in colon epithelial cells and plays an important role in the gut. Researchers think that PPAR-*γ* suppresses inflammation by regulating the aggregation of macrophages to inflammatory sites in the colon. Zhang et al. observed that curcumin inhibited trinitrobenzene sulfonic acid (TNBS) induced colitis by activating PPAR-*γ*, improved the long-term survival rate and reduced the macroscopic score of colitis in rats with IBD. They also observed that curcumin combined with dexamethasone could improve the expression of PPAR-*γ* and inhibit the expression of COX-2 and PGJ2 (Zhang et al., 2006a).

Curcumin significantly reduced the expression of CD4+T cell subsets and B and T Lymphocyte Attenuator (BTLA) in DSS induced UC mice. The mechanism of its action may be that it inhibits Th1/Tc1 cell differentiation by regulating the balance between CD4+/CD8+T lymphocyte subsets. These processes promote upregulation of BTLA expression on the surface of CD4+T lymphocytes and decrease of immune response, thus exerting anti-inflammatory and therapeutic effects on UC (Zhang et al., 2015).

#### 3.1.3 Curcumin’s Molecular Targets in IBD

The treatment of IBD focuses on controlling inflammation, thereby improving symptoms. At present, the clinical effect of anti-inflammatory or immunosuppressive drugs is not ideal. Curcumin is considered as a potential treatment for IBD due to its significant anti-inflammatory effect and safety. Curcumin may mediate anti-inflammatory effects through the following targeting molecular pathways.

Curcumin controls inflammation by downregulating the genes associated with oxidative stress and fibrogenesis pathways. The activity of PI3K and phosphorylation of AKT will result in decreasing cell death. Meanwhile, curcumin blocks neutrophils and downregulates the phosphorylation of PI3K and AKT (Ghoneim et al., 2002).

Signal transduction pathways play a crucial role in a cascade of inflammatory and are considered as potential molecular targets for the treatment of IBD. P38 mitogen-activated protein kinase (MAPK), a key regulatory enzyme of multiple target genes, regulates intestinal inflammation by regulating monocyte infiltration and intestinal water and electrolyte balance. The activation of MAPK system can lead to the large expression of inflammatory mediators, resulting in intestinal inflammatory damage. Studies have shown that curcumin can significantly inhibit p38MAPK activation and histone acetylation in intestinal mucosal cells cultured *in vitro* from children and adults with IBD, thereby reducing inflammatory responses (Epstein et al., 2010b). Similarly, curcumin can reduce the release of TNF-*α* and other pro-inflammatory factors by inhibiting p38MAPK signaling pathway, thus alleviating intestinal mucosal injury and symptoms in DSS induced IBD mice (Yang et al., 2013). Li et al. induced IBD model mice with dextran sodium sulfate (DSS) to explore the mechanism of curcumin in the treatment of IBD. The results showed that the expression of p38MAPK protein and mRNA was significantly decreased in curcumin treatment group, and the production of TNF-*α* was also decreased. In another experiment, curcumin reduced apoptosis and alleviated acetic acid induced colitis injury in rats by modulating the p38MAPK and JNK pathways (Topcu-Tarladacalisir et al., 2013).

Curcumin can inhibit the antigen presentation and maturation of dendritic cells by down-regulating the ability of spleen dendritic cells to express costimulatory signals, thus reducing the secretion level of pro-inflammatory factors. The infiltration of inflammatory cells was then inhibited, and the inflammatory damage of intestinal cells was relieved (Yang M. et al., 2017).

**FIGURE 3:**
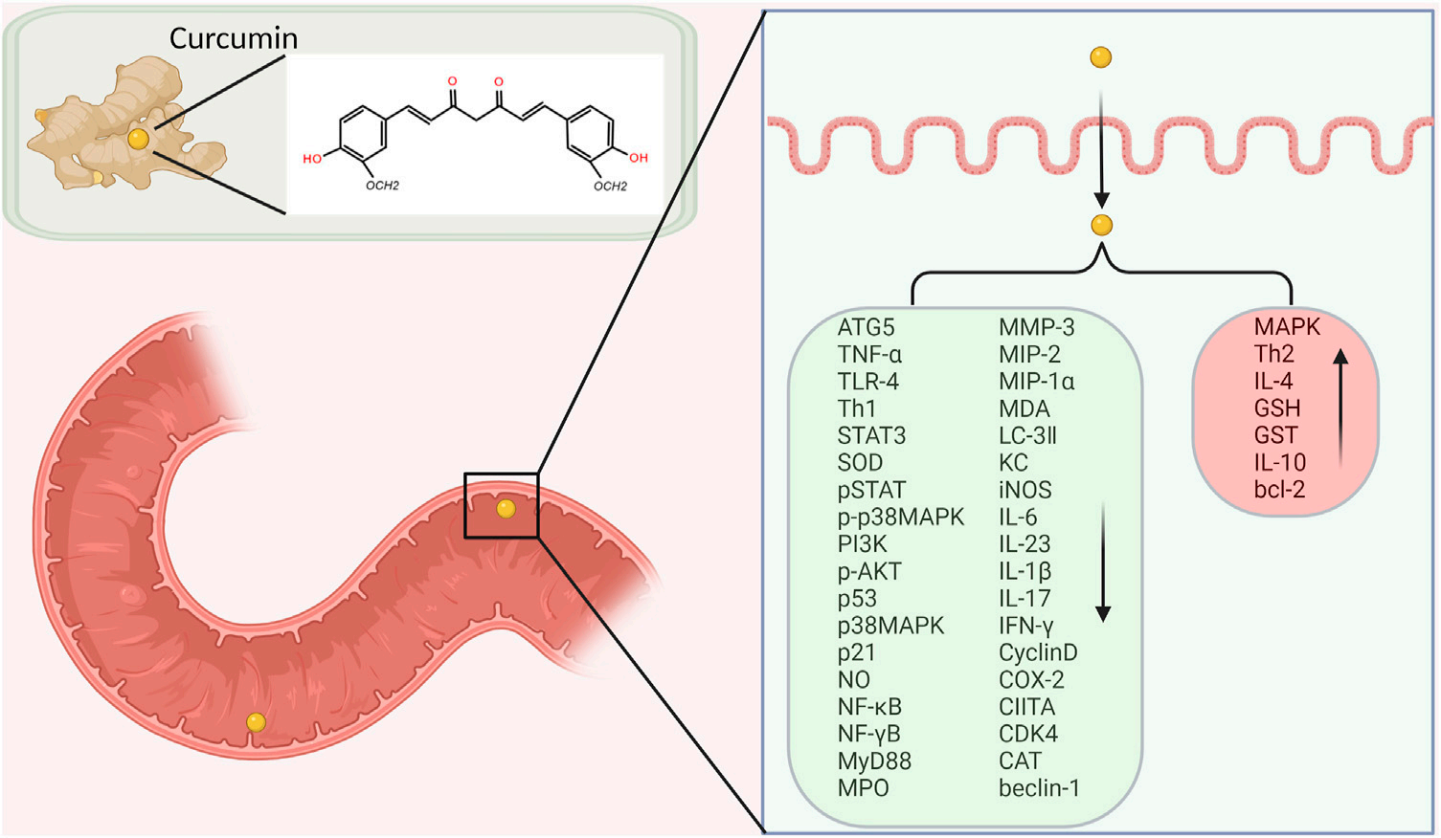
Curcumin’s molecular targets in inflammatory bowel disease (IBD) treatment.

At present, it is generally believed that intestinal mucosal immune imbalance is one of the main pathogenesis of IBD. Many proinflammatory cytokines, such as IL-1, IL-6 and INF-*γ*, promote inflammatory responses through the JAK/STAT pathway. Studies have found that STAT3 phosphorylation state is the highest in the STAT family in UC and CD patients and DSS induced IBD model mice, suggesting that STAT3 may play an important role in the pathogenesis of IBD (Suzuki et al., 2001). Curcumin exerts anti-inflammatory effects in DSS-induced IBD mice by inhibiting the STAT3 pathway (Liu et al., 2013). Yang et al. proved that curcumin could inhibit p53 expression by inhibiting STAT3 signaling. P53 is an upstream regulator of the CDK4-cylind1 complex, and therefore the levels of cell cycle regulators CDK4 and Cylind1 are decreased accordingly. Curcumin thus had an anti-inflammatory effect, and reduced the severity of DSS induced colitis in mice (Yang et al., 2013). Yue et al. ‘s experiments confirmed that curcumin can prevent DSS-induced colitis by inhibiting excessive autophagy and regulating subsequent cytokine related pathways (Karthikeyan et al., 2021) (Figure 3).

#### 3.1.4 Effects on Immune Cells

IBD is an autoimmune disease involving both autoantibodies and autoreactive CD4+T lymphocytes. Both UC and CD are characterized by immune response to intestinal antigens, but their immune response patterns are different. The pathogenesis of CD is mainly related to the inflammatory response dominated by interleukin (IL-1), IL-6, IL-8, tumor necrosis factor (TNF -*α*) and interferon *γ* (IFN-*γ*), which are secreted by Th1 and Th17 cells. UC is associated with inflammatory responses dominated by cytokines such as IL-4, IL-5, IL-9 and IL-13, which are secreted by TH-2 cells (Shale et al., 2013; Vecchi Brumatti et al., 2014; Sreedhar et al., 2016). The results of Zhang et al. showed that curcumin decreased the expression of Th1 cytokines in colonic mucosa, and at the same time up-regulated the expression of Th2 cytokines. Curcumin also upregulated the proportion of IFN-*γ*/IL-4 in the circulation. By regulating the balance of Th1/Th2, the effect of IBD treatment can be achieved (Zhang et al., 2006b).

#### 3.1.5 The Antioxidant Stress Effect

The activity of Myeloperoxidase (MPO) in mice with colitis was significantly reduced after curcumin administration. Therefore, oxidative stress, cytokine cascade and colitis response can be partially reduced (Camacho-Barquero et al., 2007). Shukla et al. demonstrated that after taking curcumin, the level of superoxide dismutase (SOD) in serum of rats increased, indicating that curcumin has good antioxidant and anti-inflammatory effects. Curcumin can eliminate oxygen free radicals in cells, inhibit lipid peroxidation in tissues, and protect SOD activity in tissues, thus playing an anti-inflammatory role in the treatment of ulcerative colitis (Shukla et al., 2003). Curcumin also plays a protective role in ulcerative colitis by regulating the antioxidant/antioxidant balance (Arafa et al., 2009) (Tables 2, 3).

**TABLE 2:** Details of curcumin’s role in inflammatory bowel disease treatment in animal studies.

Experimental colitis	Dose (mg/kg)	Duration	Effects on BW/DAI/cell apoptosis etc.^a^	Effects on biomarkers	Ref.
IL10^−/−^ mice	8–162	20 weeks	Survival rate↑ colon weight/length ratio↓ bacterial richness↑ age-related decrease in alpha diversity↓ the relative abundance of Lactobacillales↑ Coriobacterales order↓	—	McFadden et al. (2015)
TNF-*α*-induced colitis in mice	25	6.5 h	colon oxidative stress and neutrophils influx↓ cell apoptosis↓ macroscopic and microscopic colon damage scores↓	MPO↓MDA↓	Mouzaoui et al. (2012)
TNBS-induced colitis in rats	100	5 days	BW↑ macroscopic and microscopic colon damage scores↓ inflammation grade↓	TLR-4↓MyD88↓NF-*γ*B↓MPO↓MDA↓	Lubbad et al. (2009)
DSS-induced colitis in mice	36.8–92	7–14 days	the severity of DSS-induced colitis↓	STAT3↓CyclinD↓CDK4↓p53↓p21↓	Yang et al. (2013)
Acetic acid–induced colitis in rats	100	12 days	Colon inflammation score↓ BW↑ macroscopic and microscopic colon damage scores↓	MPO↓MDA↓SOD↓CAT↓MAPK↑	Topcu-Tarladacalisir et al. (2013)
DSS-induced colitis in mice	50	7 days	DAI↓ macroscopic and microscopic colon damage scores↓	pSTAT↓MPO↓IL-1β↓TNF-*α*↓	Liu et al. (2013)
TNBS-induced colitis in rats	30	14 days	BW↑ macroscopic and microscopic colon damage scores↓	MPO↓Th1↓IFN-*γ*↓Th2↑IL-4↑	Zhang et al. (2006b)
TNBS-induced colitis in rats	50–100	14 days	BW↑ macroscopic and microscopic colon damage scores↓	MPO↓TNF-*α*↓COX-2↓iNOS↓p38MAPK↓	Camacho-Barquero et al. (2007)
DSS-induced colitis in rats	100	7 days	Relative colonic weight/length ratio↓ macroscopic and microscopic colon damage scores↓	TNF-*α*↓MPO↓NO↓MDA↓GSH↑GST↑	Arafa et al. (2009)
DSS-induced colitis in mice	15, 30, 60	7 days	DAI↓ macroscopic and microscopic colon damage scores↓	TNF-*α*↓MPO↓p-p38MAPK↓p38MAPK↓	Li et al. (2015)
DSS-induced colitis in mice	15, 30, 60	7 days	DAI↓ macroscopic and microscopic colon damage scores↓	TNF-*α*↓IL-6↓IL-17↓ATG5↓LC-3Ⅱ↓ beclin-1↓autophagosome↓IL-10↑bcl-2↑	Yue et al. (2019)
DSS-induced colitis in mice	15	7 days	DAI↓ macroscopic and microscopic colon damage scores↓	MPO↓TNF-*α*↓	Beloqui et al. (2016)
DSS-induced colitis in mice	36.8–92	7 days	DAI↓ the body weight↑ the shortening of colon↓ restored the architecture of the mucosal layer↑	NF-κB↓STAT3↓COX-2↓iNOS↓	Yang et al. (2018)
DSS-induced colitis in mice	100	7 days	DAI↓ spleen index scores↓	IL-6↓IL-17↓IL-23↓IL-10↑	Wei et al. (2021)

a BW: body weight, DAI: disease activity index.

**TABLE 3:** Details of curcumin’s role in inflammatory bowel disease treatment *in vitro*.

Cell line	Dose (μM)	Effects on biomarkers	Pathways involved	Ref.
IEC-6, HT-29, CACO-2	0–150	NF-κB↓	the IκB/NF-κB pathway	Jobin et al. (1999)
Colonic epithelial cells (CEC), peritoneal macrophage, young adult mouse colonocytes cells (YAMC)	50	PI3K activity↓AKT phosphorylation↓F-actin polymerization↓MIP-2↓KC↓IL-1β↓MIP-1*α*↓	PI3K/AKT pathway	Larmonier et al. (2011)
Colonic myofibroblasts (CMF)	0–30	p38 MAPK↓NF-κB (-)MMP-3↓IL-1β↓IL-10↑	MAPK p38 pathway	Epstein et al. (2010b)
BM-derived dendritic cell (BMDC)	1–100	IL-10↑NF-κB↓IL-6↓TNF-*α*↓	NF-κB pathway	Cong et al. (2009)
T-84 cells, young adult mouse colonocytes cells (YAMC)	0–75	IFN-*γ* ↓CIITA↓	Jak-Stat signaling pathway	Midura-Kiela et al. (2012)

### 3.2 Clinical Trial

#### 3.2.1 Remission Induction

In Holt’s study, five subjects with ulcerative proctitis had received 5-aminosalicylic acid (5-ASA) before entering the study. In treatment group, researchers took curcumin 550 mg twice a day for 1 month. Then it was changed to 550 mg three times a day for 1 month. Biochemical, inflammatory factors and other indicators of subjects were examined after taking the medicine. The results showed that the clinical symptoms of all five patients improved, including two patients who had stopped taking 5-aminosalicylic acid and two patients who had reduced the dosage. Five Crohn’s disease subjects were given curcumin 360 mg 3 times a day for 1 month. Then it was changed to 360 mg 4 time a day for 1 month. The results showed an average decrease of 55% in disease activity index, 10 mm/h in erythrocyte sedimentation rate, and 0.1 mg/dl in C-reactive protein. There was no significant effect on liver and kidney function in all subjects (Holt et al., 2005).

Lang et al. enrolled patients with mild to moderate activity ulcerative colitis who did not respond significantly to the maximum dose of oral 5-ASA. They were randomly divided into treatment and control group. Both groups of patients continued to take 5-ASA orally. Patients in treatment group were given curcumin capsule (3 g/d) and patients in control group were given the same dose of placebo. The experiment lasted for 1 month. The results showed that 53.8% of patients in treatment group had symptomatic relief, while none in control group had symptomatic relief (OR = 42, 95%CI: 2.3–760) (Lang et al., 2015). Singla et al. enrolled patients with mild to moderate UC activity in the distal colon who had taken mesalazine for at least 8 weeks prior to enrollment. They were divided into treatment group and control group. The treatment group was given curcumin enema while mesalazine was taken orally, and the control group was given placebo enema while mesalazine was taken orally. The symptoms of patients in the two groups were observed. The results showed that treatment group had a higher remission rate and clinical response rate (remission rate: 43.4 vs. 22.7%, response rate: 56.5 vs. 36.4%) (Singla et al., 2014). Masoodi et al. also enrolled patients with mild to moderate UC activity. The patients were randomly divided into treatment group and control group. The treatment subjects received curcumin (80 mg, 3 times/d) and mesalazine (3 g/d), and the control group received the same dose of placebo and mesalazine. The disease severity was assessed at the end of the 4th week. The results showed that compared with the control group, the mean score of the simple clinical colitis activity index (SCCAI) in treatment group was significantly decreased (1.71 ± 1.84 vs. 2.68 ± 2.09, *p* = 0.05) (Masoodi et al., 2018).

#### 3.2.2 Remission Maintain

Hanai et al. studied the ability of curcumin to prevent UC recurrence. A randomized, double-blind, multicenter trial was conducted in 89 patients with UC at resting stage. In addition to salazopyridine or mesalazine, 43 patients received curcumin 1.0 g after breakfast and dinner, and the remaining 46 patients received placebo. The trial lasted for 6 months. Subjects’ clinical activity index and endoscopic index were assessed at the beginning of the trial, every 2 months, and at the end of the trial. The recurrence rate was 4.65% in treatment group and 20.51% in control group. The result indicated that curcumin can significantly improve colitis activity index (CAI) and endoscopic index (EI) in patients (Hanai et al., 2006) (Table 4).

**TABLE 4:** Details of curcumin’s role in inflammatory bowel disease treatment in clinical trails.

No.	Dose	Duration	Population	UC or CD	Drug combination	Main findings	Effects on serum indexes	Ref.
1	550 mg	Twice daily for 1 month, then 3 times/Day for 1 month	5	UC	5ASA, prednisone	The number of stools↓ the quality of stools↑ medication eliminated or reduced	ESR↓ CRP↓	Holt et al. (2005)
	360 mg	3 times/Day for 1 month, then 4 times/Day for 2 months	5	CD	Colestid, Flagyl, Budesonide, 6 MP	CDAI scores↓ bowel movements↓ abdominal pain↓ formed stools↑	ESR↓ CRP↓	
2	3 g/Day	1 month	50	UC	5ASA	Clinical response and remission↑ Endoscopic response and remission↑	—	Lang et al. (2015)
3	140 mg/Day	2 months	45	UC	5ASA	Clinical response and remission↑ Mucosal healing↑	—	Singla et al. (2014)
4	240 mg/Day	1 month	56	UC	Mesalamine	SCCAI↓	—	Masoodi et al. (2018)
5	2 g/Day	6 months	89	UC	5ASA, sulfasalazine, mesalamine	CAI↓ EI↓ Clinical remission↑	—	Hanai et al. (2006)
6	2 g/Day	2 months	20	UC	Selenium	DAI↓ Clinical remission↑ Endoscopic response and remission↑	ESR↓ CRP↓	Shapira et al. (2018)
7	1.5 g/Day	2 months	70	UC	—	Clinical remission↑ CAI↓	ESR↓ high-sensitivity CRP↓	Sadeghi et al. (2020)
8	360 mg/Day	3 months	30	CD	—	CDAI↓ Clinical remission↑ Endoscopic response and remission↑	—	Sugimoto et al. (2020)
9	100 mg/Day	12 months	69	UC	Mesalamine	Clinical response and remission↑ Endoscopic remission↑	—	Banerjee et al. (2021)

*5ASA, 5-aminosalicylic acid; 6 MP, 6-methylprednisone; CDAI, Crohn’s disease activity index; CAI, Clinical Activity Index; SCCAI, Simple Clinical Colitis Activity Index; EI, endoscopic index; ESR, erythrocyte sedimentation rate; CRP, C-reactive protein.

## 4 Conclusion

IBD is difficult to delay to heal and easy relapse. The economic burden of patients is heavy, and the quality of life is often seriously affected. The effect of medicinal treatment is often affected by the compliance and economic status of patients. At present, the drug effect is limited and often accompanied by side effects (i.e., diarrhea and lymphopenia). Curcumin, as a natural product with low price, has received increasing attention in recent years. Many studies have indicated that it has multiple biological activities. However, some researchers have questioned the effectiveness of curcumin. But the comment co-authored by Dr Heger and other scientists argued that the double-blind, placebo-controlled trial (DBPC) is the best way to get evidence of the medicinal value. At present, many researches have proved that curcumin is safe and effective through DBPC test (Heger, 2017). Curcumin may not fit medical chemists’ definition of the perfect drug, but many *in vitro*, *in vivo* and clinical trials have irrefutably confirmed its medicinal potential (Li et al., 2015; Beloqui et al., 2016; Yang et al., 2018; Yue et al., 2019; Wei et al., 2021). We searched ClinicalTrials.gov to identify current clinical trials evaluating curcumin treatment for IBD. A total of 10 trials were included. Three of them have been completed and five are in phase 3. We need to pay close attention to those latest findings.

Curcumin has less adverse reactions and high safety during use. As a potential chemotherapeutic drug, it shows a good application prospect in both basic experimental researches and clinical trials of IBD over the decades (Shapira et al., 2018; Sadeghi et al., 2020; Sugimoto et al., 2020; Banerjee et al., 2021). However, its mechanism of action is complex and related to multiple signaling pathways. Its mechanism of targeting has not been clearly described. More efforts are needed to identify the molecular targets and regulatory mechanisms of curcumin. Curcumin is unstable under physiological conditions and the bioavailability after oral administration is low. In order to improve its characteristics, researchers have tried various ways such as preparation of compounds, using liposomes, synthesis of nanoparticles or curcumin analogues. Some of them have yielded gratifying results (Tsuda, 2018). However, more multi-center large-scale clinical trials are needed to confirm its efficacy and safety. It is hoped that further studies on curcumin will provide new drug research directions for the treatment and remission of IBD in the near future (Li et al., 2017; Annese, 2019; Fança-Berthon et al., 2021; Rogler et al., 2021).

.

## Abbreviations

5-ASA, 5-aminosalicylic acid; BTLA, B and T lymphocyte attenuator; BW, body weight; CAI, clinical activity index; CD, Crohn’s disease; DAI, disease activity index; DCs, dendritic cells; EI, endoscopic index; DSS, dextran sodium sulfate; IBD, inflammatory bowel disease; KC, cytokine; LPS, lipopolysaccharide; MAPK, mitogen-activated protein kinase; MMP-3, matrix metalloproteinase-3; MPO, myeloperoxidase; NF-κB, nuclear factor-κB; PPAR-*γ*, peroxisomeproliferator-activated receptor *γ*; SCCAI, simple clinical colitis activity index; SOD, superoxide dismutase; Tregs, T regulatory cells; TLR-4, toll-like receptor 4; TNBS, trinitrobenzene sulfonic acid; UC, ulcerative colitis.
